# The Thirtieth Annual Meeting of the American Dental Association

**Published:** 1890-10

**Authors:** N. S. Hoff


### PROCEEDINGS.

### The Thirtieth Annual Meeting of the American Dental Association.

Reported for the Dental Register, by Dr. N. S. Hoff.

The meeting was called to order in the Opera House at Excelsior Springs, Mo., Tuesday morning, August 5th, 1890, at 11 o'clock, by President M. W. Foster. The officers present were President, M. W. Foster; 1st Vice-President, A. W. Harlan; 2nd Vice-President, J. D. Patterson; Rec. Secretary, Geo. H. Cushing; Treasurer, A. H. Fuller, and seven members of the Executive Committee.

The roll was called and the reading of the minutes of previous meeting by vote was dispensed with.

Dr. F. H. Gardiner, of Chicago, read the following resolution, and moved its adoption:

WHEREAS, There is to be a World's Columbian Exposition in Chicago in 1893; and

WHEREAS, In consequence of the fact that the choicest products of the world are to be there displayed, it is expected that citizens in large nembers of all civilized countries will be gathered together there for the purpose of seeing these exhibits; and

WHEREAS, It is to be presumed that many dentists from foreign countries will visit the United States at that time; and

WHEREAS, The time of the exposition will be an opportunity for a great meeting of dentists of the world; and

WHEREAS, It is believed that a great advance in the science and practice of dental and oral surgery would result from a meeting of the dentist of the United States with those from foreign countries who might then be visiting this country; and

WHEREAS, It is desirable that any meeting then held should be at the instance of the American Dental Association and the Southern Dental Association, and organized by a joint committee by them appointed; therefore be it

*Resolved*, That the president of this association appoint a committee of five, to confer with a like committee appointed by the Southern Dental Association at its last meeting upon this subject, and that this joint committee have power to fill all vacancies, and shall add to its membership either one, three, or five more members as it may deem advisable; and when this committee is so completed it shall be clothed with full power to take such action as it in its judgment may deem best for creating an organization for the purpose of holding a dental meeting in Chicago in 1893, which the reputable dentists thoughout the world shall be invited to attend, and that any action that this committee may take in the premises shall be final and binding.

The motion met with a prompt second, and as soon as it was stated by the president a lively discussion ensued.

Dr. Allport stated that at the last meeting of the Southern Dental Association a similar resolution was adopted, and a committee was appointed to present the matter to this association, and that committee was present and ready to present the resolution of the Southern Association, and he thought it would be decidedly discourteous to adopt the resolution of Dr. Gardiner.

After considerable discussion a motion to lay the matter on the table carried, and Dr. Carpenter, of Atlanta, Ga., was called upon to present the resolutions adopted by the Southern Dental Association. The resolutions, which were handsomely engrossed on parchment, were duly presented and read by the secretary. In substance they were the same as Dr. Gardiner's. On motion the resolutions were received and unanimously endorsed, and the president instructed to appoint a committee of five to confer with the committee from the Southern Dental Association.

The following committee was appointed: L. D. Shepard, of Boston, Mass.; W. W. Walker, of New York City; A. O. Hunt, Iowa City, Iowa; H. B. Noble, Washington, D. C.; G. W. McElhany, Columbus, Ga. The committee appointed by the Southern Dental Association is as follows: Drs. J. Y. Crawford, Nashville; L. D. Carpenter, Atlanta, Ga.; J. Taft, Cincinnati. Dr. Barton, Paris, Texas; C. E. Stockton, Newark, N. J. Elected by the joint committee: Drs. John Storey, Dallas, Texas; M. W. Foster, Baltimore, Md.; H. J. McKellops, St. Louis, Mo.; J. S. Marshall, Chicago, Ill.; A. W. Harlan, Chicago, Ill. W. W. Walker was elected Chairman of the Committee; A. O. Hunt, Secretary, and J. S. Marshall, Treasurer.

The animated discussion following Dr. Gardiner's resolution was intensified by a communication, read by the secretary, from the S. S. White Dental Manufacturing Co. The communication, which was somewhat lengthy, referred to the paper read by Dr. Louis Jack at the Saratoga meeting last year on "Independent Dental Journalism," and especially to the remarks of Dr. W. C. Barrett, who, in discussing this paper characterized the arrangement between the association and the S. S. White Dental Co., whereby that company was given the privilege of publishing in the *Dental Cosmos* the papers read before the association, to the exclusion of other journals, as "dirty work" and "managed by the politicians of the association." The communication gave at length a full history of the manner in which the agreement was brought about, the terms, etc., and asked that the matter be investigated in order that the S. S. White Dental Manufacturing Co. be cleared from the charge of underhand dealing in this matter and the integrity of the officers of the association be vindicated. A very excited discussion followed, in which it was shown that the arrangement had been entered into at a time when the publication of the proceedings caused the association considerable financial embarrassment, and was purely a business contract that had been honestly made and faithfully consummated by the interested parties, and there was no reason why any one should complain. There seemed to be a feeling that it was beneath the dignity of a scientific body to have business relations with a commercial enterprise, and that the association should control its own work, and publish its own proceedings independent of dental supply houses. A motion prevailed to have the president appoint a committee of three members of the association to investigate the matter referred to in the communication for the purpose of ascertaining the historical accuracy of the statements contained in it. Drs. W. X. Sudduth, H. A. Smith, and Geo. H. Cushing were appointed as the Committee, who subsequently reported that they found the statements were correct and that all the transactions of the S. S. White Co. had been fair and honorable, and recommended a continuation of the present arrangement for publishing the transactions. The report was, by vote, received and adopted, but at a subsequent meeting of the association the motion to adopt was reconsidered, and the part referring to a continuation of the arrangement expunged. It is reported that another supply house conducting a journal would print the papers and proceedings this year, but up to date no arrangement has been made.

The chairman of the executive committee reported the programme for the meeting. The chairman of the publication committee made a financial report, and the chairman of the committee on credentials made a partial report, all of which were adopted.

By a unanimous vote the visiting members of the Southern Dental Association were invited to participate in the discussions, and accorded the privileges of the meeting.

Resolutions of sympathy were adopted for Drs. W. H. Morgan, of Nashville, Tenn.; and E. T. Darby, of Philadelphia, both regular and honored attendants on the meetings of the association, but because of serious illness were prevented from attending this meeting.

Dr. A. W. Harlan took the chair and introduced President M. W. Foster, who read his annual address, of which the following is an abstract:

The address referred to the great variety in the dental laws of the several states. All but five of the states and territories and the District of Columbia have dental laws, each with its own peculiar requirements for admission to practice.

We have laws that compel graduates as well as non-graduates to submit to an examination ; some that recognize the diplomas of reputable dental colleges ; others that permit only graduates to be examined, excluding all non-graduates, and a few that compel all applicants, whether holding a diploma or not (except a medical diploma) to submit to an examination.

It is not just that men who have spent time and money in a regular college course should be subjected to the same conditions as those who have never taken such a course, but have gained the experience or knowledge through an office pupilage, or perhaps, may have never had either. Such laws are not only a reflection on the work of our colleges, but make it possible for men to enter with very meagre preparation, and the tendency will be to fill our profession with men who have no scientific knowledge upon which to base their practice. The laws that discriminate against the non-graduates may sometimes harm very worthy men. In this day there is not often any excuse for a man entering the profession by any other way than through the colleges, but there are many excellent men practicing to-day and highly honored in the communities where they live that would have to get a diploma to be allowed to practice in some of the states were they to remove to them. The laws that admit only graduates to their examinations, seem to favor the colleges, but in reality they cast a slur upon the diplomas of all colleges by subjecting those who hold them to a re-examination before permitting them to practice. The inference is that state examining boards are likely to have more wisdom or be more virtuous than the college faculties. Such is not likely to be the case when we consider the way in which the state boards and college faculties are selected.

The remedy is in such a unification of the various state laws as will place the granting of diplomas in the hands of a combined committee, composed of the state boards and college faculties in the various states where the colleges are located, such diplomas would then admit to practice in any state of the Union and be respected abroad.

The address recommended that the profession petition Congress to appoint dentists with suitable rank and pay to the army and navy wherever there are sufficient numbers of either to justify such an appointment.

It was suggested that the profession endorse the practice of taking impressions of the teeth of criminals for purposes of identification. It advised the establishment in Washington, D. C., of a dental headquarters with a national museum and library in connection therewith. The advantages of such a library and museum would be incalculable, and its position at Washington would give it the advantage not only of a central location, but the various museums and libraries of the national capitol would be available in many ways and possibly aid could be secured from the national government for its maintenance.

The address referred to the influence of the Association in inducing the National Faculty Association to extend the course of study in the colleges to three years. It would be well for the association to give the colleges that moral support that will make a high standard of attainment the qualification for graduation rather than a period of time study. On motion the various recommendations of the address were referred to the section on Dental Education for discussion when the section made its report.

The evening session was called to order at 8 o'clock by Presi. dent Foster. After transacting some miscellaneous business, the Treasurer's report was read showing a balance in the treasury of $1,368 77, after expending $1,739.81 during the year.

The next business was the report of sections. Sections V, VI, VII, I and II were called, and Section II, Dental Education, Literature and Nomenclature being the only one prepared to report, Dr. C. N. Peirce, the chairman, reported that Dr. Atkinson would read a paper on Education, Nomenclature and Terminology. Dr. A. H. Thompson a paper on Scientific instruction in our colleges. Dr. Rodrigues Ottolengui on How to Inaugurate a National Dental Degree. Dr. Chas. B. Atkinson on Education and the Obligation Involved and the chairman would make a report for the section.

Dr. Thompson's paper was a plea for more scientific instruction in our colleges, as opposed to what is popularly, though erroneously, called practical teaching, in which methods of practice are given preference over scientific training in the fundamental principles governing these methods. The tendency of all such teaching is to commercial rather than a scientific basis. This effort to exclude science for methods of practice that are oftentimes utterly unscientific is not education at all, and is debasing not only to the noblest qualities of the mind, but to science itself. Pure science should be taught in our colleges not only for its value in the practical realm, but for its ethical value to the students. If the practical system of education, so well exemplified in the old system of office pupilage, is the best, why not return to it and abolish the college system?

The colleges are so organized and conducted as to happily combine the scientific and industrial ideas of education, but we must not allow the industrial to consume the valuable time that a college course affords for the acquirement of a knowledge of those sciences that make the basis of our profession. The contemplated extension of the college course will provide a grand opportunity for a thorough teaching of the fundamental sciences, and the friends of true education should be watchful that the opportunity is not dissipated by allowing an undue amount of time to be devoted to the mere practical. A systematic graded course of study is now practical, which will result in a better educated class of graduates than was possible under the shorter course. The great difficulty has been that the men entering our profession have been admitted with too meager general education, and the college work has consequently been more difficult and less fruitful in high attainments. Simplification the first year, precision the second and amplification the third should be the key-note to the extended course.

There will be a demand for a new set of text-books. The first year books should be brief and simple, but sufficiently comprehensive and exact to admit of a clear statement of rudimentary science; those of the second year more ample; while those of the third should be broad and comprehensive.

The teachers should be capable and skillful, and should devote their whole time to teaching and investigation. The busy practitioner, however competent as a practitioner has no time to prepare to meet a class and impart important scientific truths in a clear and intelligible way so that the average student will be able to grasp them.

The new State laws, that compel graduates to pass an examination before they can practice in those States, may result in giving more attention to the practical branches than ought to be given, at the expense of the scientific; for these examining boards will be composed of practical men and they may not be scientific. Before such a practical board a scientifically educated man might appear at a disadvantage.

The friends of scientific education in our colleges must be on the alert that the practical man may not overload the curriculæ of the new course with practical work to the exclusion of the already difficult scientific.

Dr. Louis Ottofy read a paper written by Dr. Chas. B. Atkinson, of N. Y., on Education and the Obligations Involved.

The paper advocated the teaching of methods that were generally practiced and not the pet methods of individual teachers, on the principle that every man thinks his baby the handsomest although it may be as homely as a week old gosling. The best methods only should be taught, especially in the practical departments.

There should be uniformity and harmony in the teaching. The course should be so graded and divided that each teacher should complete his particular part, and the work of one teacher should so harmonize with that of the others as to make no confusion in the minds of the students. If this is not practicable the work should be definitely divided so there shall be no conflict of teaching.

Dr. Ottofy read a paper written by Rodrigues Ottolengui, M.D.S., of N. Y., entitled How to Inaugurate a National Dental Degree.

The writer maintains that the degree, Doctor of Dental Surgery, does not represent the standard of professional attainment that it should, owing to the facility with which the dental colleges confer it. He thinks that money is too much of a consideration in determining the qualifications of candidates for the degree. This results from the fact that the salaries of the teachers are dependent upon the size of the class. The remedy is to deprive the colleges of the right to grant diplomas and place it in the hands of a National Board of Examiners, making the present colleges merely educational. Then have a National Examining Board of twenty members, appointed under a special charter from the United States government, appointed from as many districts of the United States, who shall examine candidates and confer the special degree of Doctor of Oral Surgery. This board shall hold annual meetings and confer degrees on all candidates that are worthy, whether they have a college or office education, it matters not, so they possess the requisite knowledge. A fee of from $20 to $30 should be paid by each person taking the examination and this money should go into the treasury of the board to meet the expenses. The plan also provides for an emergency where candidates are unable to attend the annual meeting of the board, they can take their examination from the nearest member and have it acted upon at the full board meeting subsequently. The writer thinks that the new degree would in a few years become so popular that the colleges would seek to have the board examine all their men and confer this degree, and then it would be an easy and simple matter to inaugurate a National University or system of dental education. There should be no reiteration of lectures from year to year, a new set of lectures should be prepared each year or at least revised and brought up to date. In order that a better educated class of students may be secured, the paper advised that the local society appoint a committee to examine all applicants for admission to colleges in its respective territory.

---

### THE REPORT OF THE SECTION ON DENTAL EDUCATION, LITERATURE AND NOMENCLATURE.

---

BY C. N. PIERCE.

---

Three new dental colleges have been organized since the last meeting and one has disbanded. The total number of colleges now is thirty-three. Nine hundred and sixty-three students were graduated, one hundred and sixty-seven more than the previous year, a ratio of increase of three to one over the previous year. Three thousand six hundred and five students have graduated since 1885. This rapid increase in the number of graduates of the dental colleges is an aggressive menace to the profession, and it is well for the profession to see to it that these men are properly qualified, especially since it appears that any one can get a charter, organize a school and graduate men on such standards as his faculty may elect.

The report approves of the idea of establishing a nationa museum at Washington and recommends the dental profession to be on the alert to see to it that in case a national university is established at Washington, that the dental department shall be one that will represent the highest idea of professional education.

It is important that this Association give its moral support in some tangible way to those colleges that comply with the resolution of the National College Faculty Association requiring three full courses of study in separate years for graduation. The extended course of instruction will offer an opportunity to present the fundamental principles of dental science in a manner that is not possible in the present brief course, and it is now possible to establish a higher standard for graduation.

The men appointed as examiners by the States requiring an examination of all who wish to practice in them, should be well educated men and of a high order of manipulative skill, as well as of the strictest integrity. The report recommended the appointment of a committee of five to consider the proposition to establish a National Board of Examiners to confer a special degree for high attainment in dental science.

There are at present between 90 and 100 dental societies in the United States. Every State but four has a State organization and some States have several local societies. These societies have a membership of nearly 3,000 members. Last year but 22 of these societies were represented at the meeting of this Association and these societies represented only 12 States. Three Sates contributed 75 of the 175 members at that meeting. This is not as it should be; every State and every society should send a representative to this meeting and, if necessary, defray his expenses. If this was done and such delegate would present a report of the doings of his society for the year in such shape that it could be recorded under its proper section, a complete epitome of the progress of the profession for the year would be secured and could be recorded in the proceedings where it would be available for ready reference.

DISCUSSION OF THE PAPERS AND THE REPORT OF SECTION II.

Dr. H. B. Noble, Washington, D. C.—I was much interested in the recommendation contained in the president's address that the State laws be made uniform. In Washington we would be very glad to get any kind of a law, as we now have none at all. The reason is that our law must be enacted by the national Congress and it is almost impracticable for us to convince the members of Congress of the justice of such a law and get them sufficiently interested to vote for it. If there could be a uniform law enacted in a large number of States, we would have little difficulty in convincing a majority in Congress of the importance of such a law for Washington. The members of this Association can aid us in this matter by interesting their congressmen in our behalf. The law we are now trying to have enacted is one compelling every one wishing to practice in Washington to pass an examination before an examining board appointed by Congress.

I am sorry to find that this Association is no more of a representative body than the statistics of the chairman of the section indicate. If the proposition to have one special delegate from each society is feasible, it ought to receive serious consideration.

E. Parmly Brown, New York.—This question of the unification of our State dental laws is a very important one and should claim the interest of the best element in our profession. Laws that compel every man, whatever his attainments may be, or however long and honorably he may have been engaged in successful practice, to submit to an examination by a board of examiners, appointed through political influence, before he can engage in practice, are wrong and unjust and should not be sanctioned by any influential dental society. If I wished to move to New Jersey and engage in practice, I would be compelled to submit to an examination by men to whom I taught the first principles of dentistry.

C. S. Stockton, New Jersey.—They have tried for a great many years to enact a uniform marriage law and failed. If a commit tee from this Association should in five years time be able to compile a perfectly satisfactory law, it would take twenty-five times five years to get the legislatures of all the States in the Union to enact it.

Years ago in New Jersey we had no law; then we got a law compelling all non graduates to pass an examination before a State board; then we tried to get a law allowing us to confer a degree, but in this we failed and are all heartily glad of it. We now have a law that compels every man wishing to practice in New Jersey to pass an examination, whether he be a graduate or not. This law works admirably to keep out unqualified men, graduates as well as non-graduates, but is no detriment to any man who is properly qualitied. One graduate applied to the board for a permit who had a record of 100 in all his studies, but who did not know how to attach a cuspid tooth to a rubber plate and build up the inside cusp with rubber.

The method of conducting the section work of the New Jersey State Society is to appoint a person to read a paper before the society, which paper must be in the hands of the secretary three months before it is read so that some one else may be appointed and have time to write a second paper reviewing this paper and discussing the same subject.

Dr. A. W. Smith, Ky.—Did not see how the unification of the State laws could be accomplished, and if it could be, did not think it would have any tendency to secure greater proficiency in the men entering the profession.

The idea that the various societies sending delegates to this meeting should pay their expenses is a good one. It is no more than right that a man who is willing to give his time for the good of his profession should receive at least renumeration for his actual expenses.

Dr. J. Y. Crawford, Tenn.—In Tennessee we have no law to regulate the practice of dentistry. Is it not possible that the vast amount of discussion on this subject has been time wasted? Has the aggregate result advanced the standard of dental education correspondingly? An effective law must be based upon justice and equity, and because some of our laws lose sight of this great moral principle they are oppressive, unjust and do not accomplish the purpose for which they were enacted and can not be enforced. A lawyer who is admitted to practice in one State will have his papers accredited in any other; it ought to be so with the dental profession. Let us be generous, there is no danger of overcrowding the profession. If you were to equip every dentist in the United States with a set of scalers and turn them loose they could not begin to remove the tartar from peoples teeth. We need a higher standard of education, but we must not lose sight of the great underlying principles of right and justice in our efforts to obtain it.

Dr. J. B. Story, Texas.—Wanted to know of Dr. Stockton if the young man learned to attach the plain cuspid to the rubber plate as instructed, (Dr. Stockton said he did). Then I say that young man ought to have been given a permit to practice even in New Jersey. A man graduating from college with such a record and being capable of appropriating instruction in that way is not going to be kept long in ignorance of ways of mere manipulation.

We have a law in Texas that requires the judge of each of the 47 judicial districts, to appoint three competent dentists to examine candidates for admission to practice. What troubles us is to find men to serve on these examining boards as we do not have as many dentists in the whole State. And then how is a district judge to determine who is competent? All these laws are faulty from the fact that political influence will secure the appointment of unworthy men on these examining boards.

Knowledge, not time, should secure a diploma and no college that does not act upon this principle should have its diploma recognized, but all those that do should have their diplomas accepted in any State in the Union.

Dr. W. W. Allport, of Chicago.—Dr. Story stated the case fully when he said that the diploma of any reputable dental college should be accepted as a passport in any State in the Union. This would be so if all colleges graduated men on the principle of sufficient knowledge, but unfortunately all colleges are not what they should be; they are in business to make money, not to educate dentists. Hence, it becomes necessary to have State examining boards to revise their work. In the State of Illinois any man can get a charter for a dental college for three dollars. Infamous and illiterate men take advantage of this fact for the sake of the money to be made out of it.

I am heartily in sympathy with the effort being made by the dentists of Washington to have a law regulating the practice of dentistry in the District of Columbia passed, and I move that this Association request the United States House of Representatives to pass the bill that is now before it and which the Senate has already favorably acted upon. This motion was seconded and unanimously adopted.

Dr. C. L. Goddard, California.—The difficulty in the way of enacting uniform State laws lies in the fact that all these laws are the result of a process of time and evolution. The only way to unify these laws would be by national legislation. In one instance the State Board of Examiners of California admitted to practice nine men, two of whom had been rejected by the dental department of the University of California and the other seven were undergraduates.

Dr. P. T. Smith, Colorado.—Laws do not increase our moral standards. They are physical processes which hold us in check, but do not educate us morally.

The eloquent and beautiful paper of Dr. Thompson, with its luteal and soothing influence, which almost lulled us to sleep, had nothing in it to which we can object, or criticise; we can only applaud its sentiments. But the paper of Dr. W. H. Atkinson was one that will cause us to think. It not only gives us the foundation principles of all moral activity, but accounts for the inspiration that is inborn to us, enabling us to attain to higher degrees of perfection in any noble calling. It is our duty to bring to bear upon our every day calling the fundamental principles which govern our being and account for its inspiration; it is only when we do so that we are enabled to apply such agencies as will tend to perfect the highest and best attainments of which we are capable. The chemical constituents of our bodies are only the vehicles or media through which our spiritual natures are manifest, and a lack of this media or an imperfect combination will in due proportion affect the symmetry and harmony of our being. So it is in practical education, the moral as well as the scientific and practical must be considered in their proper relations in order to reach the highest standard of education.

Dr. Jas. Truman, Philadelphia.—This is a very important period in the transition of dental laws. Sometimes laws are oppressive and tyrannical. When they become so it is time to repeal them. I am not opposed to dental laws but believe in them; but at the same time our laws should be based upon justice and equity. I would favor the adoption of a uniform dental law in all the States.

Our attention has been called to the manner of imparting instruction in colleges. As an old teacher, I wish to say that a man who can not stand before his class and present his subject in clear plain language that can be readily comprehended, is no teacher and should step down and out. The fundamental principles of dental science can and should be stated in language that will command the attention of the student and fix the idea upon his memory at once. I have sometimes wished that all lecturing could be dispensed with and some practical method of imparting instruction adopted.

This Association meeting is a great educating medium and should be more universally attended. I would not miss one of its meetings for a great deal. We should not only attend the meetings of our societies and help to make them profitable, but we should patronize the literature of our science. The great work which Dr. Farrar has put so much time and money into, ought to be in the library of every dentist though it should cost him a hundred dollars, which it will not.

Dr. J. N. Crouse, Chicago.—Made a statement as to the condition of the affairs of the Dental Protective Association, and asked that a committee of four persons representing different sections of the country be appointed to examine the Dental Protective Association as to its methods of doing business, its investments, accounts, etc., and report at the next meeting.

The suggestion of Dr. Crouse was approved and the president appointed Drs. J. Y. Crawford, W. W. Walker and Frank H. Gardiner. The other member to be afterwards appointed by the president.

On motion a committee of three consisting of Drs. W. H. Atkinson, J. D. Patterson and C. L. Goddard were appointed by the president to protect the interest of the profession in the case that has been appealed from the New Hampshire Supreme Court to the Supreme Court of the United States, involving an adverse decision to the State dental law. The committee was authorized to employ counsel if thought necessary and draw upon the treasury of the Association for a sum not to exceed five hundred dollars.

EVENING SESSION, AUGUST 6TH.

Section II was passed without further discussion and Section III, Operative Dentistry, was called.

Dr. A. E. Baldwin, of Chicago, having been elected chairman because of the absence of Dr. Darby, read two papers, one by Dr. Chas. B. Atkinson, of New York, on "Medicated Oxyphosphate Fillings," and another by Dr. J. L. Williams, of Boston, Mass., on "Preservative and Obtundent Treatment of Teeth."

Dr. Atkinson's paper advocated mixing the various medicinal agents, such as: creosote and oil ot cloves, eugenol, deliquesced carbolic acid, cinnamon oil, clove oil, pure creosote, creosote oil of cloves and iodoform, creolin, campho-phenique, potassium chlorate, salicylic acid, camphor, stick sulphur pulverized, iodoform, oil of wintergreen with oxyphosphate fillings in treating teeth. The medicaments when powered are mixed with the oxide of zinc and when liquid with the phosphoric acid. The writer claims to have had excellent results from the use of a pulp capping composed of creosote, oil of cloves and oxide of zinc, covering this with a thin oxyphosphate filling to which some antiseptic remedy has been added. The writer advises the same mixing of antiseptic remedies with oxyphosphate used in setting inlays, crowns and retaining or regulating appliances.

Dr. Williams' paper advocated treating sensitive dentine and exposed pulps with soothing antiseptic non-irritating remedies sealed into the cavities by plastic fillings, removing and replacing as often as necessary until nature has had a chance to produce a healthy condition, unhindered by irritating medicines or zymotic influences. Thirty-four years of practice on this principle enables the writer to commend this practice with assurance. Advises against the use of all caustic or irritating applications. A solution of calcium chloride in deep seated caries is an excellent anti-ferment. Dr. Williams claims to have been the first to propose a treatment that would favor the deposition of secondary dentine. Also the mixing of oxide of zinc with gutta percha.

The report of the section was read by N. S. Hoff, Secretary. The report was a general review of the progress made by the operative department of dentistry as expressed in the contributions in the way of books, papers and discussions, instruments, appliances, materials, etc. The report endeavored to enumerate all the suggestions and inventions of the year, calling special attention to such as are likely to prove specially helpful. The report recommended, and the officers of the section have planned to do so next year, a division of the subject of operative dentistry into its principle departments and the appointment of one member who shall collect everything that transpires during the year in the department specially allotted to him and report it at the next meeting. Appointments have already been made on the following subjects: Treatment of children's teeth, materials used in filling teeth, operating instruments and appliances, treatment of exposed pulps, literature, and conduct of an office. Other subjects will be arranged for soon and it is hoped that the report next year will be the most complete one ever made on this subject.

Dr. C. N. Pierce—Wished to call attention to the action of peroxide of hydrogen on tooth substance. He exhibited a bottle containing one half ounce of peroxide of hydrogen into which he had placed a tooth with a large amount of exostosed substance on the end of the root. The liquid was changed every twenty-four hours for several days. Each day a large amount of white floculi or white deposit would appear at the bottom of the vial, until the exostosed portion had all disappeared, when the action upon the cementum was less rapid, the same fact was noticed also when the dentine was reached. There was no apparent roughness of the tooth that would indicate the absorption of the organic or inorganic portions, but both seem to be equally abraded. Its use then should be with caution.

Dr. T. W. Brophy.—It is well known that sulphuric or hydrochloric acid are used to keep the peroxide of hydrogen stable. Dr. Pierce's experiment would be more satifactory if he had ascertained before using whether the peroxide was of an acid or neutral reaction. The experiment would have been more conclusive if the peroxide had been known to be neutral in its reaction. It is well to discriminate in the use of these strong drugs.

Dr. M. L. Rhein, of New York.—Have obtained results similar to this in dissolving salivary calculus with the peroxide.

Dr. A. W. Harlan, Chicago.—The action of this drug on the tooth has received too little attention on the part of the profession. Peroxide of hydrogen of 12 per cent. solution should be absolute ly neutral, but I have only been able to secure two varieties that were so, namely, Tromesdorf's and Schuchard's.

Dr. D. R. Stubblefield, Nashville.—I do not think sulphuric acid is ever used in the preparation of peroxide of hydrogen. Hydrochloric acid is used in its manufacture and this acid would produce the result Dr. Pierce has obtained.

Dr. Wm. Conrad, St. Louis.—Dr. Fisher read a paper at the last meeting of the Missouri Dental Society in which he stated that a prominent manufacturing chemist of St. Louis, told him that sulphuric acid was added to peroxide of hydrogen to secure its stability. (No acid is used in its manufacture according to Mitchell's Chemistry *Reporter*.)

Dr. W. H. Atkinson.—When I first used peroxide of hydrogen for cleaning the debris out of root canals, I thought I had found my washerwoman, but I soon found that the bubbling resulted in the presence of other things, blood and pus as well as tooth substance. Glycozone is preferable as it is a more reliable preparation than peroxide of hydrogen.

The peroxide containing the tooth was examined with test papers by Dr. Harlan and found to have a decidedly acid reaction. He was of the opinion that the acid was hydrochloric.

Dr. Louis Ottofy, Chicago.—I have employed medicaments in composition with oxyphosphate for filling over sensitive dentine and pulp capping with good results.

Dr. Story, Texas.—I do not believe much in capping exposed pulps with any sort of a remedy, don't use many remedies anyway. Oxychloride of zinc is good enough germicide and disinfectant for me.

I capped a pulp two years ago and it was all right for a while, but one day the patient came in with a swollen face. I removed the filling, cleaned out the pulp canals and filled with oxychloride of zinc and have had no trouble since.

In another case I filled a tooth with amalgam over a nearly exposed pulp, and after a few years the tooth gave trouble and on removing the filling I found the pulp had receded and died. I don't need a great lot of fine instruments for introducing my oxychloride into the root canals. I find a piece of whalebone worked down to the proper size to admirably answer my purpose.

Dr. E. Parmly Brown, New York.—Don't let two dead pulps frighten you off from saving two hundred alive. A tooth with a live pulp in it is worth a dozen dead ones. The idea of incorporating medicaments with oxyphosphate dressing is a good one. I use South Sea Island asbestos incorporated with oxyphosphate as a dressing and capping for exposed pulps, it prevents thermal irritation and is non-irritant.

Dr. P. T. Smith.—Pulps die not so much from irritating fillings as from starvation.

Dr. Story.—I generally find that where pulps have died after filling, that the teeth were very sensitive when filled and something was used to relieve the sensitiveness.

Dr. J. N. Crouse.—The record of my cases of capping exposed pulps is different from Dr. Story's. I occasionally have a failure but it is the exception and not the rule. I believe the operation of capping exposed pulps is a successful one when properly done. The difference in results in my operations is that of skillful manipulation and bungling.

Dr. Story.—Dr. Crouse lives in Chicago and I live in Texas; he can save pulps in Chicago but I can't do it in Texas; I don't practice dentistry to make trouble for my patients or myself either. I have from two to three funerals in my office every week.

Dr. Crawford.—I think the trouble is that we don't discriminate in the selection of cases. You can't save a pulp or successfully cap it when it is all gone except a small portion in the root canals, but you can successfully cap many nearly exposed or accidentally exposed pulps, or those largely exposed in strong and robust patients.

Dr. Baldwin.—In this practice we must take into consideration the climacterical condition of the patient. There are certain periods in the lives of patients when all surgical and remedial efforts are likely to fail. The local pathological condition, the physical condition of the patient and the environments, climate, etc., are important matters of consideration, but all the conditions should be recognized and provided against if we expect to be successful. The condition of the patient is everything. We may think we have succeeded often in saving exposed nerves, when if we open the tooth we will find dead pulps. It is not my practice to attempt to save a pulp that has been subjected to pulpitis. It makes considerable difference, too, whether a pulp has been actually exposed.

Dr. Rhein.—I think we ought to endeavor to save the pulps of every young and growing tooth.

Dr. Conrad.—I deem it very proper to attempt to save the pulps in young and growing teeth, but it is in my opinion, sure death to apply medicinal agents mixed with oxyphosphate to exposed pulps.

Dr. A. W. Harlan.—When I was in Europe last year I had an opportunity to inspect some specimens of white rubber inlays, such as were referred to in the section report. The fillings looked very much like the teeth and they are easily made and inserted and take a very good polish. I see no reason why they should not be used to good advantage in many places.

Dr. A. J. Swasey, Chicago.—I have seen some of this white rubber inlay work, but the only rubber inlay that I have seen that fitted the cavity snugly was made of black rubber. I use a gold inlay which is made by shaping the cavity as for the ordinary inlay, beveling the border slightly. Into this is burnished approximately a sheet of No. 120 gold foil, then a piece of eras-ing rubber is cut to about the size and shape of the cavity and placed in over the gold and the patient asked to close the teeth together thus forcing the rubber down into the cavity and caus-ing the gold to conform to the exact shape of the cavity; a burnisher is passed around the edge and the gold brought up close to the margin of the cavity; the gold is then removed and trimmed to nearly the right size, annealed and replaced in the cavity; the rubber is again put in place and an additional piece of rubber somewhat larger than the cavity put over it, and the patient again requested to close the teeth upon it; this will cause the gold matrix to adapt itself perfectly to the cavity; remove from the cavity and invest in one-third hard coal ashes and two-thirds plaster; melt into this matrix twenty karat gold using a large flame with a blow-pipe. The sides of the inlay are grooved with a file and the surface roughly finished, and the inlay is set with a thin cement, using the mallet to gradually tap it to place; when set, finish as if an ordinary gold filling.

MORNING SESSION, THURSDAY, AUG. 7TH.

Section V, Materia Medica and Therapeutics.—Dr. Harlan, the chairman of this section, in lieu of a formal report, read a paper entitled "Acidum Carbolicum."

The paper gave an historical sketch of the discovery of carbolic acid and its therapeutical application. A new kind of carbolic acid has recently been put on the market, it is called synthetic carbolic acid and is manufactured in Germany. It is clear, crystalline, with a slight odor which does not resemble the coal tar product. Is soluable in twelve parts of water making a clear solution. A five per cent. solution has no perceptible odor. The germicidal action is about the same as pure ordinary carbolic acid, but neither of these possess as great a germicidal activity as a mixture of crude carbolic acid and sulphuric acid. Carbolic acid should not be used in arsenical preparation for destroying pulps, as the pulps and tooth will degenerate more rapidly, use instead oil of cinnamon, cloves or cassia. Carbolic acid should not be used in the treatment of roots for the reason that it coagulates the organic structure and does not penetrate the dentine, thus leaving zymotic influences to start a fermentative and disintegrating process after the tooth has been filled, changing its color and perhaps precipitating its destruction. Its action on a blind abscess is to convert it into an acute one. It should never be used in the treatment of diseased gums uncombined with other drugs that will neutralize its causticity; it may be combined with camphor, tannin, glycerine or some of the oils. It should never be applied to a pulp that is to be capped, as its caustic action is fatal. Astringents and soothing remedies should be applied to exposed pulps. It should never be used on cotton to be left permanently in roots as it readily decomposes and possesses no embalming quality. Copal, damar, Canada balsam and agents of this class may be used for this purpose. It should never be used for the treatment of abscesses through the roots of teeth; permanganate of potash, silico fluoride of sodium solution, oil of cloves, cassia, cinnamon and agents of this class are preferable as they are diffusible and will not clog the dentinal tubuli and are better antiseptics.

The only desirable uses of carbolic acid are in the form of a spray, as a local anæsthetic, component of mouth washes, or in combination with other remedies for the treatment of diseased gums and medication around the roots of teeth.

There being no discussion of this subject the next section was called.

Section VI, Physiology and Etiology.—Dr. H. A. Smith, Cincinnati, read the report of the section. The subject of implantation is being followed up closely in keeping statistics of cases. It seems that some of those who have been interested in this subject have lost confidence in its permanent results, as many of what were formerly reported successful operations have since proved failures.

The report stated that the matter of tabulating the prehistoric crania of the country, which was referred to this section last year to be done, was to be taken up at once. Difficulty had been experienced in adopting a satisfactory system of classification. Dr. J. J. R. Patrick, of Illinois, has consented to undertake the work and his system of classification would be adopted. The report also referred with commendation to the work of Dr. E. G. Betty, of Cincinnati, who had critically examined all the crania of the Army Medical Museum at Washington, tabulated the results and published them in the *Dental Review* of April, 1890.

Dr. Patrick read his plan for tabulating the statistics and explained his ideas as to what we should seek to establish in this investigation. Blank forms of the plan will be printed and may be had of Dr. Patrick. It is desirable that every person having access to a collection of such crania, however small it may be, should apply for these forms and make the examination and return the result to Dr. Patrick for tabulation and classification. The dental profession owes a great deal to a few men who have spent their time, money and lives in investigations for the benefit of dental science. Here is a chance for a great many to unite in a grand work that will be productive of great good to our science.

Dr. W. S. How, Philadelphia, read a paper on "Hillischer's system of notation and indices." This paper was interesting, but an abstract of it without the tables would not be sufficiently intelligible or valuable to warrant us in attempting it in this report. It will be published with the transactions.

Dr. L. Ottofy stated that the average per cent. of successful operations in implanting teeth had been because of additional failures, reduced from seventy-five to sixty per cent. Reported a case where an implanted tooth had been cut off at the neck by absorption and the root remained intact. Dr. M. H. Fletcher, of Cincinnati, had experimented by implanting teeth in a goat's leg and jaw and finds upon killing the animal that there was absorption, more or less, in every case.

Dr. Goddard showed a specimen of tooth prepared for implantation operations. It was a natural root to which a Logan crown was attached. The joint was made by a gold filling entirely around the root.

Dr. H. A. Smith recommended the adoption of an international system of notation. The French and Germans already have one, but what we need is one that will be adopted and used everywhere. This is a subject that could be very happily adjusted at the great international meeting in 1893.

Dr. Ottofy suggested that it would be well for the Association to adopt a resolution inviting visitors to the meeting of 1893 to come prepared to discuss this question and submit a system of international notation for consideration.

Section VII, Anatomy, Pathology and Surgery was next called and Dr. M. L. Rhein read the report, which called attention to the fact that surgical operations that were peculiarly within the province of the dentist to perform, were not attempted by him at all, but if brought to his notice, they were at once referred to the general surgeon for treatment.

The reason for this was largely due to the fact that dentists were ignorant of the anatomy of the facial region and of the pathological conditions involved and could not carry out a systematic treatment of cases. Our education is at fault here. We need more and better text books on these subjects and this branch of instruction needs revising.

Dr. Rhein read a paper written by Dr. Arthur C. Hugenschmidt, of Paris, France, entitled "Occasional Origin of Alveolar Abscess from Teeth with Living Pulps."

The paper related three cases in proof of the assertion that abscess can occur and the pulp remain alive. Two of the cases were molar teeth and one a central incisor. When the abscesses appeared the teeth were drilled and manifested all the sensations of teeth with living pulps. The drilling was discontinued and treatment applied to the abscesses externally without successful results. The pulps were then exposed, destroyed and the abscesses yielded to ordinary treatment. The paper attributes the cause of the abscess in these cases to the irritation of a filling which resulted in a hypertrophied condition of the pulp, which produced absorption of the dentine and cementum at the end of the root and consequently enlargement of the foramina. The inflammatory condition of the pulp is conveyed by continuity of tissue outside the tooth canal to where the hard tissue is less resistant and the inflammation becomes localized into an alveolar abscess.

The doctor concludes that a chronic alveolar abscess does not always arise from a devitalized tooth, but a living tooth may be the only source and origin of true alveolar abscess. And when such an abscess does occur from a living tooth there is necessarily enlargement of the apical foramina.

Dr. M. L. Rhein read another paper written by himself on "The Amputation of Roots as a Radical cure in Chronic Alveolar Abscess, Complicated with Pyorrhœa Alveolaris."

This important operation receives little or no notice from the text books or the profession generally. It presents no anatomical or surgical difficulties. The only instruments needed are a sharp spear-head drill to drill through the process and root at a right angle to the canal, and a new sharp fissure bur that will cut laterally to sever the root. Generally no anæsthetic will be required. Cocain may be successfully used, and if the operation is likely to prove tedious and painful, it would be well to have a physician administer chloroform or ether. If there is an area of dead bone surrounding the end of the root it should be burred out with a large bur.

If antiseptic precautions are observed and the root cut off smooth and clean, there will be no question as to the result of this operation. The doctor instanced three extremely bad cases for which he had successfully operated. The roots were drilled, cut off and removed, the dead bone burred away, the cavity washed out with a solution of bichloride of mercury in peroxide of hydrogen and the wound was allowed to heal under iodoform dressings. This operation is especially applicable to the roots of upper molar teeth. It is applicable to all teeth.

EVENING SESSION.

Dr. Taft, of the committee of necrology, presented resolutions on the death of Dr. Homer Judd, of St. Louis, which were unanimously adopted.

On motion of Dr. Truman, the place for holding the next meeting was left to the committee of arrangements.

The president declared the next order of business to be the election of officers and stated that an informal vote would be taken. This resulting in the election of Dr. A. W. Harlan by 60 votes out of a total of 71. By a unanimous vote this ballot was made a formal one and Dr. Harlan was declared duly elected president for the ensuing year.

The following were duly elected to the respective offices:

J. D. Patterson, first vice-president; H. B. Noble, second vice-president; Geo. H. Cushing, recording secretary; Fred. A. Levy, corresponding secretary; A. H. Fuller, treasurer; C. N. Pierce, H. A. Smith and L. D. Shepard, members of the executive committee.

After the election, the president asked Drs. Gardiner and Walker to conduct the newly elected president to the platform. President Foster, in a graceful speech, thanked the members of the Association for the courtesy shown him during the meeting and asked that the same considerate treatment might be extended to the newly elected president, Dr. A. W. Harlan, whom he was pleased to present to the Association as its presiding officer for the ensuing year.

Dr. Harlan, in a brief speech, thanked the members of the Association for the great honor they had conferred upon him. When I entered this Association nineteen years ago, I had no idea I should ever become its presiding officer. I ask your indulgence and hope we may all work together to make the meeting of this Association next year the best in all its history.

A resolution was unanimously carried commending Excelsior Springs as an admirable place for holding scientific conventions.

Dr. A. O. Hunt, Iowa—gave by the aid of the micro-lantern an exhibition of photo-micrograph slides, illustrating sections of jaws and teeth, to show the manner of the blood and nerve supply of the teeth. The doctor claims that there is no evidence to show that the blood vessels and nerves pass directly from the larger main trunks in the jaw directly to the foramina at the end of root and thence into the pulp. He stated that in over fifty dissections he was unable to trace a nerve or artery directly. He believes that the vessels and nerves break up in small branches which pass through the alveolar process in various directions and enter the peridental membrane, and from this the pulp obtains its nerve and blood supply.

Dr. W. X. Sudduth, Minneapolis,—gave a lantern exhibition of slides representing the tissues found in all the varieties of tumors found in the mouth.

Both of these lectures were exceedingly interesting, but because of the darkness in the room we could secure no notes, and if we could it would be difficult to make them intelligible without the illustrations. This is one of the places where the man who will not attend the society meetings, because he can get it all from his journal, gets left.

MORNING SESSION, FRIDAY.

In order to allow section I to report, the papers read under sections IV and VII were passed without discussion.

Dr. W. B. Ames, secretary of section I, reported new appliances, calling special attention to Mathew's combination pliers for crown work and his power blow-pipe; also to Floyd's crescent teeth, which have special points of merit. Also a new form of porcelain crown with the pin baked into it, similar to the Logan crown, invented by Dr. E. Parmly Brown. The pin, instead of being slotted as the Logan pin is oval, made of platino-irridium, and roughened or threaded so as to cause the cement to adhere more firmly to it. The pin is larger at the joint than elsewhere rendering it less liable to break off.

The report presented two papers, one by Dr. C. S. Case on crown work, and another by Dr. Stubblefield on "Amalgamation and Dental Amalgams."

Dr. Case's paper described a new method of making a Richmond crown with porcelain face. For the incisor and cuspid teeth, the root is cut off and trimmed as for the old style wood pivot teeth and a collar is made in the usual way, except that it is made as broad as the crown of the tooth is long. An oval section is then cut out of the labial and palatal surfaces leaving a cone-shaped projection from the band to the mesial and distal sides of the cutting edge of the crown; into this collar a thin gold plate is burnished and soldered. It is then fitted to the root and if desired, the pin for the root adjusted, and the porcelain crown ground to place, backed for soldering as in the ordinary Richmond crown; these are then cemented with hard wax, removed from the root, invested and just enough solder used to attach the pin and porcelain front to the collar and cap. An impression of the lingual surface of a corresponding natural tooth is then obtained in mouldine and a die made upon which a gold face is swedged, which is fitted and soldered to the lingual side of the crown. This surface can be made with solder at the time that the pin and porcelain front are soldered to the collar as in the Richmond method. But Dr. Case claims not only an economy of material by his method, but a more natural and artistic result, with very little more time and labor.

For bicuspids and molars a collar is made as for the all gold Richmond crown. The buccal face of this is cut out in an oval section, but not quite detached at the center of the face from the collar; this section is trimmed and bent over on the end of the root as far as the pulp canal and soldered to the collar, making a shoulder upon which the porcelain face is fitted and soldered, in the same manner as for the incisor teeth. If required, a pin into the root can be soldered to place at the same time. The top of this crown is then ground down to allow for the metallic cap, which is swedged and soldered in place in the usual way.

This method of making a bicuspid or molar crown with a porcelain front has decided advantage over other methods, in economy of material, time and labor required, and the facility with which a pin may be used for the root canals. It it also as strong and artistic as any.

Dr. Case instead of using platinum to line the porcelain facing for the purpose of causing the solder to close up the joint, uses jewelers' white enamel, which fuses when the crown is soldered and makes an impervious joint. It is also useful in gold plate work for closing the joint between the plate and tooth and backing. The material may be obtained from any wholesale jewelery house, and needs only to be wet with water and placed in the joint before soldering.

DR. D. R. STUBBLEFIELD READ A PAPER ON AMALGAMS AND AMALGAMATION.

Mercury uniting with other metals forms an amalgam, or a true chemical compound. Unlike most chemical combinations no heat is evolved when mercury unites with zinc, lead, tin, iron, and nickel, but when combined with sodium, potassium, and cadmium heat is evolved. Mercury unites with metals of the alkali in definite proportion, forming definite compounds, but it is impossible to give a formula in the case of the complicated dental alloys used for filling teeth.

Experiments prove tin amalgams to be the best conductors of heat and bismuth the worst. In all cases lessening the amount of mercury used increases the conductivity accordingly. Amalgamation produces chemical compounds which exhibit variable and uncertain results when examined physically. Amalgam alloys probably do not dissolve into a molecular solution but to a crystalline, the crystals of which are pressed close together, and after the excess of mercury is forced out a solid crystalloid mass results.

Tin, silver, gold, platinum, and zinc all unite with mercury to form amalgams of variable qualities, but no amalgam made with any of these metals alone is useful for filling teeth. Tin amalgam is too soft and expands in setting; silver amalgam sets slowly and also expands; gold amalgamates with difficulty unless heat is applied and is too soft for use; platinum amalgam does not harden although the presence of platinum in an alloy quickens the hardening process; copper does not amalgamate readily except when finely divided, or as obtained by precipitation from its salts; zinc amalgam is too brittle for use, but zinc in an alloy is a very useful ingredient.

By mixing these various metals in proper proportions, in order to get the peculiar virtue of each metal, a useful amalgam alloy is obtained. In the manufacture of alloys great care must be exercised in order to obtain an alloy with definite proportions of metals used. Care should also be exercised in mixing with the mercury, as many excellent alloys will be ruined by an improper amount of mercury.

The writer prefers to use alloys containing zinc, as they are not likely to change form, and do not darken so much as other alloys. He also recommends amalgam as an excellent filling for root canals, and does not think copper amalgam possesses any advantages over the alloys. For the reason that he does not believe in the formation of a copper salt under the filling, to accomplish which, there must be solution of the filling, and if solution subsequent destruction must result.

